# Therapeutic Notes

**Published:** 1899-10-07

**Authors:** 


### THERAPEUTIC NOTES.

One of the difficulties which stands in the way of the use of intra-muscular injection of mercurials is that they are apt to be painful, at least for a time. It is stated, however,[1] that if a small amount of guaiacol be added to the oil used the process is rendered practically painless. The following formula is given :—

℞. Hydrarg. iod. rubr. gr. 8.
Guaiacol pur. minim. 45.
Ol. olivæ ster. fl. oz. 3.

The injections are practised daily, or every other day, 30 minims of the above mixture, representing about gr. $\frac{1}{6}$, of the biniodide being a dose. The fluid is injected perpendicularly into the muscle, the buttock being the site usually chosen.

[1] New York Med. News, Sept. 9.

